# Diagnosed Mild Cognitive Impairment Due to Alzheimer’s Disease with PET Biomarkers of Beta Amyloid and Neuronal Dysfunction

**DOI:** 10.1371/journal.pone.0066877

**Published:** 2013-06-14

**Authors:** Shizuo Hatashita, Hidetomo Yamasaki

**Affiliations:** Neurology, Shonan-Atsugi Hospital, Atsugi, Japan; Nathan Kline Institute and New York University School of Medicine, United States of America

## Abstract

The aim of this study is to identify mild cognitive impairment (MCI) due to Alzheimer’s disease (AD) using amyloid imaging of beta amyloid (Aβ) deposition and FDG imaging of reflecting neuronal dysfunction as PET biomarkers. Sixty-eight MCI patients underwent cognitive testing, [11C]-PIB PET and [18F]-FDG PET at baseline and follow-up. Regions of interest were defined on co-registered MRI. PIB distribution volume ratio (DVR) was calculated using Logan graphical analysis, and the standardized uptake value ratio (SUVR) on the same regions was used as quantitative analysis for [18F]-FDG. Thirty (44.1%) of all 68 MCI patients converted to AD over 19.2±7.1 months. The annual rate of MCI conversion was 23.4%. A positive Aβ PET biomarker significantly identified MCI due to AD in individual MCI subjects with a sensitivity (SS) of 96.6% and specificity (SP) of 42.1%. The positive predictive value (PPV) was 56.8%. A positive Aβ biomarker in APOE ε4/4 carriers distinguished with a SS of 100%. In individual MCI subjects who had a prominent impairment in episodic memory and aged older than 75 years, an Aβ biomarker identified MCI due to AD with a greater SS of 100%, SP of 66.6% and PPV of 80%, compared to FDG biomarker alone or both PET biomarkers combined. In contrast, when assessed in precuneus, both Aβ and FDG biomarkers had the greatest level of certainty for MCI due to AD with a PPV of 87.8%. The Aβ PET biomarker primarily defines MCI due to AD in individual MCI subjects. Furthermore, combined FDG biomarker in a cortical region of precuneus provides an added diagnostic value in predicting AD over a short period.

## Introduction

Mild cognitive impairment (MCI) has a multitude of causes, including predementia of Alzheimer’s disease (AD) and non-AD as well as depression and various physical disorders. A recent study has shown that subjects with MCI are at increased risk of developing AD and that their overall rate of progression to AD is typically 10%−15% per year [1]. Identifying subjects with MCI who are most likely to decline in cognition over time is a major focus in AD research. Revised research criteria for the diagnosis of AD have been proposed, and a framework has been developed to capture the earliest stage of AD [2]. Recently, the National Institute on Aging (NIA) - Alzheimer’s Association working group has proposed the diagnostic criteria for the symptomatic predementia phase of AD, referred to a MCI due to AD [3]. The MCI due to AD could be used to identify individuals with AD pathophysiological processes as the primary cause of their progressive cognitive dysfunction.

The clinical research diagnosis of MCI due to AD is based on the Core Clinical Criteria used in all clinical settings without the need of highly specialized tests and the Clinical Research Criteria with the use of biomarkers. Some biomarker measurements directly reflect the pathology of AD, including positron emission tomography (PET) amyloid imaging and cerebrospinal fluid (CSF) beta-amyloid (Aβ) 42, while other biomarkers reflect downstream neuronal injury associated with AD, including CSF tau (both total tau and phosphorylated tau), fluorodeoxyglucose (FDG)-PET imaging and MRI. The evidence of both Aβ deposition and neuronal injury together provide the greatest probability that the AD pathophysiological processes are present. A number of studies have compared the predictive value of two or more biomarkers at a time but findings have been inconsistent [4]–[6]. Some aspects of the clinical research criteria may be revised, as these criteria are put into practice and new findings become available.

The PET amyloid imaging with a tracer of [11C]-labeled Pittsburgh Compound-B ([11C]-PIB:*N*-methyl-[11C]2-[4′-methylaminophenyl]-6-hydroxybenzothiazole), which has a high affinity for fibrillar Aβ, is a reliable biomarker of underlying AD pathology [7], [8]. On the other hand, a FDG-PET imaging for cerebral glucose metabolism, reflecting downstream neuronal injury, provides evidence about cognitive function and progression that can not be provided by amyloid PET imaging. We have recently demonstrated that a diagnostic framework with an Aβ deposition by [11C]-PIB PET, in different clinical stages of AD, allows for an earlier and more specific AD diagnosis [9]. In addition, our preliminary study with FDG-PET imaging showed that cerebral glucose metabolism starts to decrease in prodromal AD, and greater decrease correlates with greater cognitive impairment along the continuum from prodromal to AD dementia.

The present study was conducted to define the MCI due to AD using PET amyloid imaging of Aβ deposition and FGD-PET imaging of neuronal dysfunction as a biomarker. We sought to determine whether episodic memory impairment, age and apolipoprotein-E (APOE) genotype have an effect on progression to AD dementia.

## Materials and Methods

### Subjects

Sixty-eight Japanese participants were recruited from our memory clinic. All were between 50 and 90 years of age. They underwent neurological and neuropsychological assessment, and neuroimaging at baseline and once more after 12–24 months. Global cognitive status was assessed with the Mini-Mental-State Examination (MMSE) [10] and severity of dementia was rated on the Clinical Dementia Rating (CDR) scale [11]. The CDR sum of boxes (CDR SB) score was a simple sum of the score obtained in each of the 6 rated domains. A memory measure of immediate and delayed recall of a paragraph from the Wechsler Memory Scale-Revised (WMS-R) Logical Memory II was carried out as a simple episodic memory test [12]. The apolipoprotein E (APOE) genotype was determined from venous blood samples.

All 68 MCI patients met the Core Clinical Criteria for MCI, proposed by the NIA-Alzheimer’s Association workgroup [3], including concern about a change in cognition, impairment in one or more cognitive domains, preservation of independence in functional abilities, and no dementia. Other systemic or brain diseases that could account for the decline in cognition, including degenerative, vascular, depressive, traumatic, medical comorbidities, or mixed disease were excluded. The MMSE score was greater than or equal to 24, and global CDR score was at least 0.5 in the memory domain. For the episodic memory measure, delayed recall of a paragraph from the WMS-R Logical Memory II (maximum score 25) was used with the following cutoff scores based on education: these scores were less than 8 for subject with ≥16 years of education, 4 for subjects with 8–15 years of education, and 2 for subjects with 0−7 years of education [12]. AD diagnosis was based on the criteria of the National Institute of Neurological and Communicative Disorders and Stroke-Alzheimer’s Disease and Related Disorders Association (NINCDS-ADRDA) [13].

Each subject or their caregiver provided written informed consent for participation. The study protocol was approved by the Institutional Review Board of the Mirai Iryo Research Center Inc (Tokyo, Japan).

### PET imaging

All PET scans were performed on the same day as the cognitive testing, using a Siemens ECAT ACCEL scanner in 3-dimensional scanning mode, providing 63 contiguous 2.46-mm slices with a 5.6-mm transaxial and a 5.4-mm axial resolution. All imaging data were reconstructed into a 128×128 matrix. The amyloid PET imaging was accomplished with the radiotracer [11C]-PIB, as previously described in detail [9]. Briefly, [11C]-PIB was produced in our PET center using the one-step [11C] methyl triflate approach. [11C]-PIB was injected intravenously as a bolus with a mean dose of 561.5±11.2 MBq (n = 68). Dynamic PET scanning was performed for 60 minutes according to a predetermined protocol. Sixty minutes following the completion of [11C]-PIB image, the subjects were injected intravenously with 249.9±28.8 MBq (n = 68) of [18F]-FDG and remained in a dark, quiet room. Fifteen-minute static FDG-PET scans were acquired with the same camera after a 45-minute uptake period, and the image was reconstructed using the same image reconstruction techniques.

### Image analysis

All subjects underwent T1-weighed MRI (1.5 T) for co-registration with the PET images. MRI-based correction of PET data for partial volume effects was carried out using the PMOD software (PMOD Technologies Ltd., Adliswil, Switzerland). Regions of interest were manually drawn on the co-registered MR image, including the following cortical regions: lateral temporal cortex, medial temporal cortex, frontal cortex, occipital cortex, parietal cortex, sensory motor cortex, anterior cingulate gyrus, posterior cingulate gyrus, precuneus cortex and cerebellar cortex.

### Data management

Levels of PIB retention were determined by the distribution volume ratio (DVR) with Logan graphical analysis for 35 to 60 minutes with cerebellar gray matter as reference [14]. Regional DVR values of each cortical region and the cortical DVR value of the whole cortical regions were calculated. DVR PET images were created for visual inspection with a rainbow color scale. Scans of all subjects were visually assessed with 2 readers as amyloid-positive or amyloid-negative for cortical PIB retention. Amyloid-positive images showed greatly increased PIB retention in the cortex compared to white matter and a typical PIB pattern of cortical amyloid deposition, whereas amyloid-negative images showed no PIB retention in any of the cortical regions.

The same co-registration method was applied for quantification of [18F]-FDG. A standardized uptake value (SUV) of the same region was obtained and subsequently normalized to the cerebellar cortex as reference. Glucose metabolism was referred to as the SUV ratio (SUVR).

### Statistical analysis

Group differences were evaluated with analysis of variance (ANOVA), followed by Bonferroni post hoc tests to assess the significance. Analysis correlation between DVR values, FDG SUVR values, age, MMSE scores, CDR SB scores and WMS-R recall scores yielded Pearson’s product moment correlation coefficient (*r)*. Categorical variables were examined with Fisher’s exact test. Paired *t*-tests were used to study changes between baseline and follow-up data. Results were considered significant at *p*<0.05. Data are presented as means ± standard deviations (SD). Statistical analyses were performed with Statcel 3 software (OMS Inc. Japan).

## Results

### Clinical data and cognitive function

Thirty (44.1%) of the 68 patients with MCI converted to AD during the follow-up of 19.2±7.1 months (converters), while the remaining 38 (55.9%) did not progress to AD (stable patients). Twelve (50%) of 24 MCI patients aged 75−89 years converted to AD, compared to 18 (40.9%) of 44 MCI patients aged 50−74 years. Fourteen (50%) of 28 APOE ε4 carriers with MCI and 16 (40%) of 40 APOE ε4 non-carriers converted to AD. In particular, 5 (83.3%) of 6 APOE ε4/4 carriers with MCI converted to AD. Overall rate of MCI progression to AD was 23.4% per year.

The demographic characteristics of MCI converters and stable patients at baseline and follow-up are summarized in Table 1. The MMSE and CDR SB scores in MCI converters at baseline did not differ from those in stable patients. The mean MMSE score of MCI converters decreased to 22.8±2.5 at follow-up and their CDR SB score increased to 2.5±1.0. The mean MMSE score of MCI converters decreased approximately 4 points whereas that of stable patients remained unchanged. There were no significant differences in age or sex between MCI converters and stable patients. None of the patients with MCI reverted to a normal status or converted to a non-AD dementia.

**Table 1:** Demographic Characteristics of Patients With MCI.

	Baseline		Follow up	
Characteristic	Converter	Stable	Converter	Stable
**study popu**	30	38	30	38
**Education (yr)**	12.2±2.4	12.0±2.0	12.2±2.4	12.0±2.0
**MMSE**	26.5±1.5	27.3±1.6	22.8±2.5*	27.3±1.6
**global CDR**	0.5	0.5	0.6±0.2	0.5
**CDR SB**	0.8±0.2	0.8±0.3	2.5±1.0*	0.7±0.4
**Immediate Rec.**	4.9±2.7	6.9±3.1	4.0±2.8	7.2±3.9
**Delayed Rec.**	1.7±2.0*	4.6±3.4	1.5±2.3	4.9±4.3
**APOE ε4/4, ε4/3**	14 (46.6%)	14 (36.8%)	14 (46.6%)	14 (36.8%)

MCI: mild cognitive impairment, MMSE: Mini-Mental State Examination, CDR: Clinical Dementia Rating, CDR SB: Clinical Dementia Rating sum of boxes score, study popu: number of patients in the study population, yr: years. Rec: WMS-R recall scores, APOE: apolipoprotein E, Data are presented as means ± SD, * Statistically significant difference from stable patients by multiple comparisons post hoc tests (*p*<0.05).

For the memory measure of WMS-R Logical Memory II Immediate and Delayed Recall, the mean delayed paragraph recall score at baseline was 1.7±2.0 for converters, which were significantly lower than the mean score for stable patients despite their having approximately the same level of education (Table 1). However, there was no significant difference in the mean immediate paragraph recall scores between MCI converters and stable patients.

### Aβ deposition

**Figure 1:**
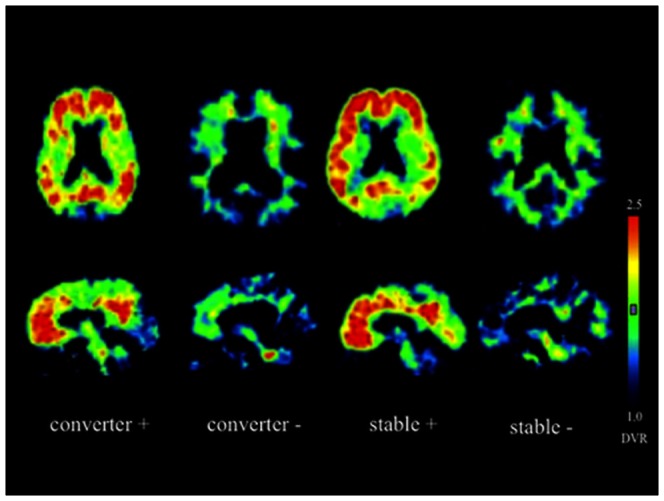
PIB-PET DVR images from 4 representative MCI converters and stable patients with (+) and without amyloid deposition (−) at baseline.

Fifty-one (75%) of the 68 MCI patients had marked PIB retention in the frontal, parietal and lateral temporal cortical regions as well as the cingulate gyrus and precuneus, showing the typical AD pattern at baseline (Figure 1). Of 51 MCI patients who had Aβ deposition, 29 (56.8%) patients converted to AD during the follow-up period while the remaining 22 patients did not convert despite similar Aβ deposition. In contrast, 16 of 17 MCI patients who had no PIB retention in any cortical region were stable and only 1 MCI patient converted to AD.

**Figure 2:**
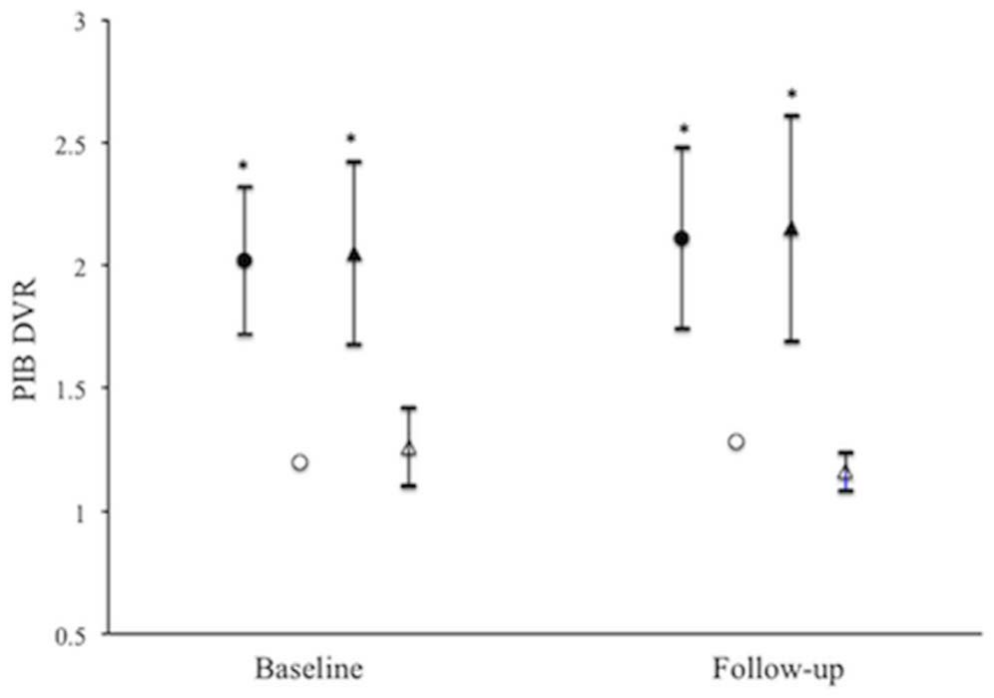
The cortical PIB DVR values in PIB-positive MCI converters (closed circles, n = 29), PIB-negative MCI converter (open circles, n = 1), PIB-positive stable MCI patients (closed triangles, n = 22), and PIB-negative stable MCI patients (open triangles, n = 16) at baseline and follow-up. Data are presented as mean ± SD. *Statistically significant difference from the PIB-negative stable MCI patients by multiple comparisons post hoc tests (*p*<0.01).

Cortical PIB DVR values for MCI converters and stable patients are presented in Figure 2. At baseline, the mean value of cortical DVR in amyloid-positive converters (2.02±0.30, n = 29, p<0.01) was significantly higher than that in amyloid-negative stable patients (1.26±0.16, n = 16). In the amyloid-positive stable patients, in addition, the mean cortical DVR value (2.05±0.37, n = 22) was similar to amyloid-positive converters. Also, there were no significant differences in the regional DVR values of any cortical region between amyloid-positive converters and stable patients. The mean value of cortical DVR in amyloid-positive converters at follow-up (2.11±0.37, n = 29) was not significantly different from baseline (Figure 2). There were no significant changes in cortical DVR values between baseline and follow-up in amyloid-positive or amyloid–negative stable patients.

### Glucose metabolism

**Figure 3:**
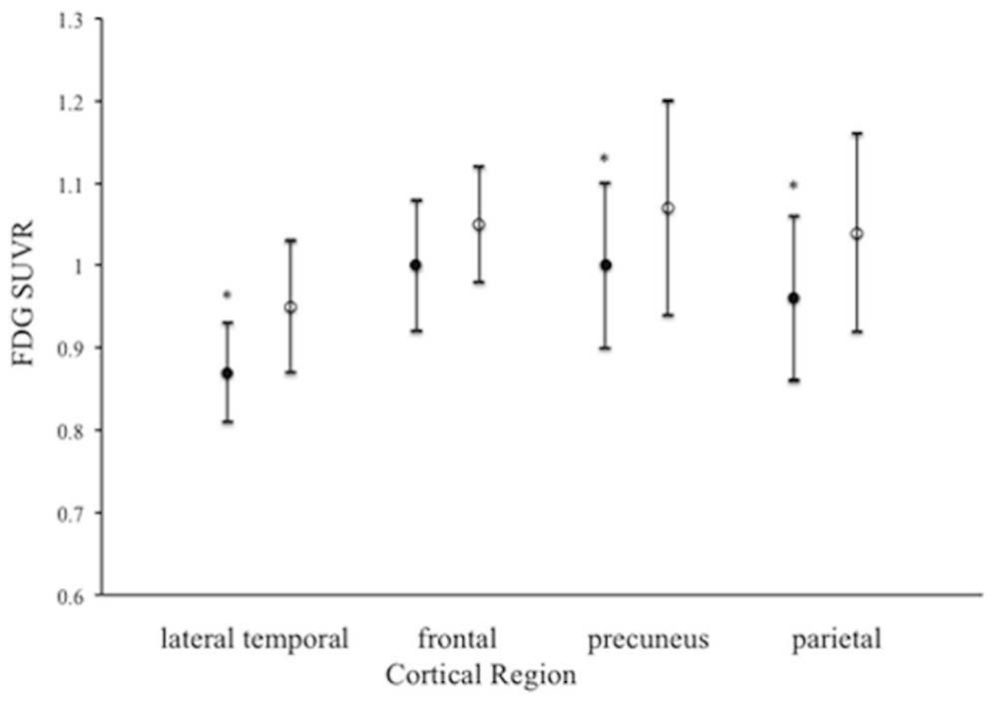
Regional FDG SUVR values in the lateral temporal cortex, frontal cortex, precuneus and parietal cortex of MCI converters (closed circles, n = 30) and stable patients (open circles, n = 38) at baseline. Data are presented as means ± SD. *Statistically significant difference from the stable patients by multiple comparisons post hoc tests (p<0.05).

Fifty-seven (83.8%) of 68 MCI patients had reduced glucose metabolism (FDG SUVR ≤0.99) in whole cortical regions at baseline, 28 patients (49.1%) of whom progressed to AD during the follow-up period. A mean FDG SUVR value for the whole cortical regions in converters was 0.93±0.06 (n = 30, p<0.01) at baseline, significantly different from stable patients (0.98±0.06, n = 38). In cortical regions of the lateral temporal cortex, parietal cortex and precuneus, in particular, MCI converters had significantly lower regional FDG SUVR values at baseline compared to stable patients (Figure 3). At follow-up, MCI converters did not have significant decreases in mean values of FDG SUVR in whole cortical regions or each cortical region.

### Comparison between Aβ deposition and glucose metabolism

Individual cortical FDG SUVR values were not correlated significantly with PIB DVR values in all MCI patients at baseline (r = –0.22, p = 0.06, n = 68). Also, there was also no significant correlation between regional FDG and DVR values in the lateral temporal cortex, frontal cortex, parietal cortex or precuneus.

### Diagnostic value of PET biomarkers

**Figure 4:**
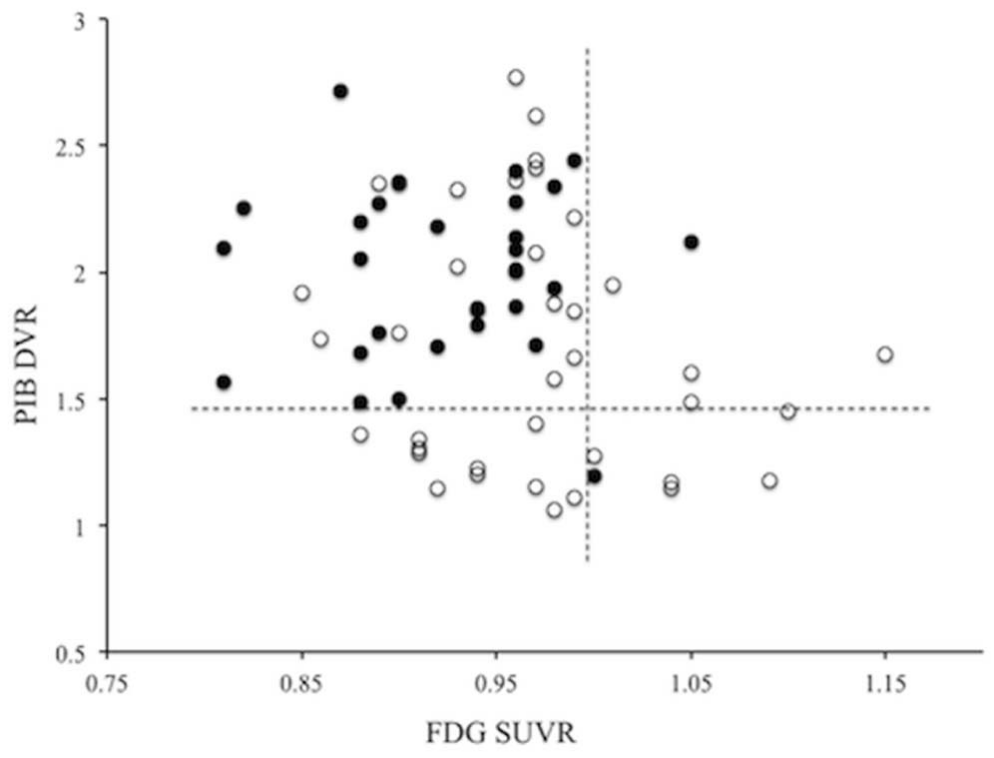
Scatter plot of the relationship between cortical PIB DVR and FDG SUVR values in individual MCI converters (closed circles, n = 30) and stable patients (open circles, n = 38). **The horizontal dotted line indicates the greatest value of PIB DVR (≥1.49).** The vertical dotted line indicates the lower value of FDG SUVR (≤0.99).

The relationships between individual cortical PIB DVR and FDG SUVR values for all 68 MCI patients are shown in Figure 4. Individual PIB DVR values for 29 (96.6%) of 30 converters and 22 (57.8%) of 38 stable patients exceeded the lowest value of amyloid-positive subjects (DVR ≥1.49), which is used as a positive Aβ PET biomarker of Aβ deposition. On the other hand, individual FDG SUVR values were below the lower value (FDG SUVR ≤0.99), which is used as positive FDG PET biomarker, for 28 (93.3%) of 30 converters and 29 (76.3%) of 38 stable patients. Positive or negative value fell within the reliable range, as previously demonstrated [9], and standardization of biomarkers for both PET amyloid imaging and FDG-PET imaging was possible in our clinical setting.

We used a dichotomous test using chi-square analysis to assess the sensitivity (SS) and specificity (SP) of positive Aβ PET and FDG PET biomarkers for MCI due to AD (Table 2). A positive Aβ PET biomarker significantly identified MCI due to AD in individual MCI subjects with a sensitivity of 96.6% and a specificity of 42.1%, compared to negative Aβ PET biomarker (p<0.01). The positive predictive value (PPV) was 56.8%. A positive FDG PET biomarker had a sensitivity of 93.3% and a specificity of 23.6%. Combined positive Aβ and FDG biomarkers did not significantly discriminate MCI due to AD in individual MCI subjects.

**Table 2:** Sensitivity and specificity of positive Aβ and/or FDG PET biomarkers for MCI due to AD in individual MCI patients.

Biomarker	SS	SP	PPV	NPV
**aged 50**–**89**				
** Aβ PET**	96.6	42.1	56.8	94.1
** FDG PET**	93.3	23.6	49.1	81.8
** Aβ+ FDG PET**	96.5	21.7	60.8	83.3
**aged ≥75 years, lower delayed recall**				
** Aβ PET**	100	66.6	80.0	100
** FDG PET**	91.6	44.4	68.7	80.0
** Aβ+ FDG PET**	100	60.0	84.6	100
**APOE ε4 carriers**				
** Aβ PET**	100	28.5	58.3	100
** FDG PET**	100	21.4	56.0	100
** Aβ+ FDG PET**	100	18.1	60.8	100

SS: sensitivity, SP: specificity, PPV: positive predictive value, NPV: negative predictive value, Aβ: β-amyloid, MCI: mild cognitive impairment, APOE: apolipoprotein E, Data are presented as percentages.

In individual MCI subjects with APOE ε4, a positive Aβ PET biomarker significantly discriminated MCI due to AD (SS = 100%, SP = 28.5%) in addition to individual APOE ε4 non-carriers (SS = 93.7%, SP = 50%), compared to negative biomarker. In particular, a positive Aβ PET biomarker in the individual MCI subjects with APOE ε4/4 distinguished with a sensitivity of 100% and a specificity of 0%. Combining Aβ and FDG PET biomarkers did not improve their sensitivity and specificity in individual APOE ε4 carriers and non-carriers.

When individual MCI patients, aged 75−89 years, had lower WMS-R Logical Memory (delayed recall score ≤4), a positive Aβ PET biomarker had greater sensitivity 100% and specificity 66.6% at identifying MCI due to AD (p<0.01), and the positive predictive value was 80%. However, combining positive Aβ and FDG PET biomarkers did not improve the discrimination of MCI due to AD compared to positive Aβ PET biomarker alone. In contrast, when assessed in a cortical region of precuneus, a combination of positive Aβ and FDG PET biomarkers discriminated MCI due to AD from individual MCI subjects with the greatest sensitivity of 96.6% and specificity of 73.3% (P<0.01). The combination had a higher positive predictive value of 87.8%.

## Discussion

We demonstrated that 44.1% of 68 MCI patients converted to AD during a follow-up of 19.2±7.1 months and the rate of MCI conversion was 23.4% per year. A recent study has reported that 48% of 31 subjects with MCI converted to AD during a follow-up period of 2.9 years [15]. The Alzheimer’s Disease Neuroimaging Initiative has reported that 32.9% of 85 single-domain or multiple-domain amnestic MCI patients converted to AD during a follow-up period of 1.9±0.4 years (an annual rate of 17.2%) [1]. Thus, conversion rates vary greatly depending on the criteria applied, the nature of the subject population and the period of observation.

In the present study, 50% of APOE ε4 carriers with MCI converted to AD while 40% of APOE ε4 non-carriers with MCI converted. This supports that the progressive value of genetic assessment alone is comparatively low although a high percentage of progressive MCI patients carry the APOE ε4 allele [18]. In contrast, this study showed that 83.3% of APOE ε4/4 carriers with MCI converted to AD. These findings indicate that the presence of the APOE ε4/4 in MCI patients is strongly associated with conversion to AD. The homozygous carriers of the APOE ε4 allele could have an increased risk of MCI progression to AD.

The present study demonstrated, using [11C]-PIB PET imaging, that 56.8% of 51 MCI patients with Aβ deposition converted to AD over 2 years, compared to 5.8% of 17 MCI patients without Aβ deposition. In addition, when MCI patients with Aβ deposition, aged 75−89 years, had an impaired episodic memory, the rate of MCI conversion was 80%. It has previously been reported that 82% of PIB-positive amnestic subjects with MCI converted to AD over a 3-year follow-up period [15], and 67% of MCI subjects with high PIB retention converted over 2 years [16]. These findings indicate that a high proportion of MCI patients with Aβ deposition convert to AD within a short period. In vivo detection of Aβ deposition with PET imaging is a useful prognostic tool for identifying MCI progression to AD.

We found that MCI converters had the regional hypometabolism in cortical regions of lateral temporal cortex, parietal cortex and precuneus at baseline compared to stable patients. Our results are consistent with the previous study that regional metabolic reduction, in temporoparietal or posterior cingulate cortices, was detected at baseline in MCI patients who converted to AD within 1 year, compared with non-converters [17]. The AD pathophysiological process has been demonstrated to be temporoparietal and/or precuneus hypometabolism, which is a topographic pattern characteristic of AD. Therefore, if a regional hypometabolism in the temporal, parietal and/or precuneus cortices is detected on FDG PET imaging, the progression to AD may occur within a few years.

An evaluation in predictive accuracy by combining different biomarkers and neuropsychological variables for the prediction of AD in MCI has been reported [19]. Inconsistencies in recent findings are likely due to a variability in neuroimaging processing techniques, CSF protein immunoassay techniques, setting cutoff values for subject categorization, study design, and statistical analysis. To determine MCI due to AD, the biomarker in diagnosis reflecting Aβ and downstream neuronal injury has been proposed to be most important [3].

In the present study, we applied PET biomarkers to diagnose MCI due to AD in individual MCI subjects. An Aβ PET biomarker identified MCI due to AD with a sensitivity of 96.6% and a specificity of 42.1%. In individual APOE ε4/4 carriers with MCI, in particular, the Aβ PET biomarker distinguished MCI due to AD with greater certainty. Furthermore, when MCI subjects had a prominent impairment in episodic memory and aged older than 75 years, an Aβ PET biomarker provided a further increased level of certainty with a sensitivity of 100% and a specificity of 66.6%, compared to FDG PET biomarker alone or both PET biomarkers combined. A recent meta-analysis has shown that sensitivity and specificity of PIB-PET for predicting clinical progression to AD range from 83.3–100% and 41.7–76.8%, respectively [20]. These findings indicate that the sensitivity of an Aβ PET biomarker is high in the prediction of conversion to AD while the specificity is low. The Aβ PET biomarker is primarily required for diagnosing MCI due to AD in individual MCI subjects.

Furthermore, in this study, a positive FDG PET alone or combined positive Aβ and FDG biomarkers did not significantly discriminate MCI due to AD in individual MCI subjects. Although a FDG PET biomarker has been associated with the neuropathology of AD, it is not specific for AD. In contrast, when assessed in the precuneus with regional hypometabolism, both Aβ and FDG PET biomarkers had a greater level of certainty for MCI due to AD with the greatest sensitivity of 96.6% and specificity of 73.3%. The combination had the highest positive predictive value of 87.8%. We suggests that most individuals with a diagnosis of MCI due to AD, using Aβ and FDG PET biomarkers in a cortical region of precuneus, progress to AD even within a short period.

The MCI due to AD could be more certainly diagnosed in individual MCI subjects, based on core clinical criteria, with the conjugation of biomarkers of PET amyloid imaging and FDG-PET imaging. The clinical research diagnosis of MCI due to AD would have the greatest potential benefit for assessing risk level in single patients before the onset of the disease, providing a basis for earlier therapeutic and pharmacologic intervention, patient counseling, and the design of clinical trials.
